# Effects of motor restrictions on preparatory brain activity

**DOI:** 10.1007/s00221-021-06190-w

**Published:** 2021-08-25

**Authors:** L. Sperl, J. M. Ruttloff, G. G. Ambrus, J. M. Kaufmann, R. Cañal-Bruland, S. R. Schweinberger

**Affiliations:** 1 Department for the Psychology of Human Movement and Sport, Institute of Sports Science, Faculty of Social and Behavioural Sciences, Friedrich Schiller University Jena, Jena, Germany; 2 Department of General Psychology and Cognitive Neuroscience, Institute of Psychology, Faculty of Social and Behavioural Sciences, Friedrich Schiller University Jena, Jena, Germany; 3 Department of Biological Psychology and Cognitive Neurosciences, Institute of Psychology, Faculty of Social and Behavioural Sciences, Friedrich Schiller University Jena, Jena, Germany

**Keywords:** Interference control, Response inhibition, Motor restriction, Immobilization, Motor skill change, Typing

## Abstract

Modifying established motor skills is a challenging endeavor due to proactive interference from undesired old to desired new actions, calling for high levels of cognitive control. Motor restrictions may facilitate the modification of motor skills by rendering undesired responses physically impossible, thus reducing demands to response inhibition. Here we studied behavioral and EEG effects of rule changes to typing in skilled touch-typists. The respective rule change—typing without using the left index finger—was either implemented per instruction only or with an additional motor restriction. In both groups, the rule change elicited delays and more errors in typing, indicating the occurrence of proactive interference. While stimulus-locked ERPs did not exhibit prominent effects of rule change or group, response-locked ERPs revealed that the time courses of preparatory brain activity preceding typing responses depended on the presence of motor restriction. Although further research is necessary to corroborate our findings, they indicate a novel brain correlate that represents changes in inhibitory response preparation induced by short-term motor restrictions.

**Supplementary Information:**

The online version contains supplementary material available at 10.1007/s00221-021-06190-w.

## Introduction

Modifying a well-established, pre-existing behavior or skill is usually difficult as interference effects may impede this change process (Panzer 2002; Sperl and Cañal-Bruland 2020a). Often, proactive interference arises due to automatisms that are prone to trigger a habitual but undesired response. As a consequence, individuals may experience response conflicts, posing particularly high demands on cognitive, and especially inhibitory, control processes (Levy and Anderson 2002; Radvansky 2017; Underwood, 1957).

Since behavioral changes often encompass the motor system, the examination of *motor* skill changes provides a particularly relevant and insightful testbed. In this domain, especially *prepotent response inhibition* has recently been reported to play a critical role in change processes (Sperl et al. 2021a, b; Sperl and Cañal-Bruland 2020b). Prepotent response inhibition is defined as a subdimension of inhibition denoting the ability to suppress strong prepotent, but undesired action tendencies (Friedman and Miyake 2004).

To empirically investigate interference effects arising from pre-existing, procedural motor skills, Sperl and colleagues recently established a novel experimental paradigm (see e.g., Sperl and Cañal-Bruland 2020b, c; Sperl et al. 2021b). Specifically, they confronted participants with different types of rule changes that disrupted their highly automatized typing skill. Typing reflects a well-automatized motor skill which is mastered by many individuals (Logan 2018) and involves a daily amount of practice that is often comparable with that of expert athletes or musicians (Kalfaoğlu et al. 2018; for other empirical paradigms, see also, e.g., Logan and Crump 2009; Snyder and Logan 2013). Besides showing strong and immediate proactive interference effects, visible in significant performance declines (measured via typing times and errors), these paradigms usually also reveal high interindividual variability in the success to deal with the new rule change.

In various studies, Sperl and colleagues scrutinized the nature of these differences and observed that lower scores in the Stop-Signal Task (a common cognitive test to measure response inhibition abilities; Friedman and Miyake, 2004) were associated with more interference in typing (Sperl and Cañal-Bruland2020b; Sperl et al. 2021b). Assuming that interference from previously established action patterns provides one of the main challenges for motor skill change, the successful suppression of dominant, but undesired response alternatives seems critical for overcoming interference (Baxter et al. 2004; Panzer, 2002). The role of response inhibition for successful interference control in motor tasks was further corroborated by a recent EEG study by Sperl et al. (2021a) who investigated ERPs associated with successful interference control (also applying the reported typing paradigm) and observed an electrophysiological pattern that was highly typical for response inhibition processes, visible in increased P3 amplitudes^1^ (see also Huster et al. 2013; Krämer et al. 2011; Xie et al. 2017). In typing research, inhibitory processes have also been observed in terms of increased pre-response positivity over the ipsilateral motor area, hence the area controlling the contralateral hand, which has been interpreted as inhibition of the currently non-relevant hand (Pinet et al. 2015; Vidal et al. 2003; for further approaches investigating LRP, see also Logan et al. 2011; Scaltritti et al. 2018).

According to Bernstein (1967), performing a goal-directed action is related to numerous degrees of freedom and mastering redundant degrees of freedom is critical for successful movement co-ordination. The motor domain offers a unique opportunity to unconventionally suppress irrelevant or undesired behavior, that is via motor restrictions. Inducing constraints may reduce motor degrees of freedom, thereby eliminating the physical option to perform an undesired motor response. While understudied in basic experimental research, constraints play a role in applied research. In medicine, injured joints are often treated by immobilization, following the logic that it is difficult to cognitively supervise permanent avoidance of a respective body part and that immobilization devices reduce the need for continuous cognitive control (cf. Diday-Nolle and Reiter Eigenheer, 2019). Importantly, constraint-induced movement therapy with motor restriction to *unimpaired* extremities has well-documented and substantial beneficial effects on cortical plasticity and motor rehabilitation of the impaired extremity in stroke patients (Wolf et al. 2006). Furthermore, motor restrictions are often used to improve technical features in the context of sport (e.g., Cotterman et al. 2005) or ergonomics, for instance, when orthopedic or robotic devices are designed to prevent individuals from unhealthy postures and to provide motor guidance (Bettany-Saltikov et al. 2008; Carrozza et al. 2019; for an extended overview on the idea of motor restriction, see also Sperl, 2021).

While previous studies already investigated the neural effects of limb immobilization on motor imagination or execution—often in terms of excitability or neural plasticity (e.g., Facchini et al. 2002; Garbarini et al. 2019; Huber et al. 2006; Ngomo et al. 2012)—little research has been dedicated towards the effect of motor restrictions on *inhibition* so far. Sperl and colleagues recently conducted two behavioral studies using the reported typing paradigm by applying a finger bandage that physically precluded the movement of a to-be-avoided finger. They observed tendencies towards a potential positive effect on inhibition processes, visible in less performance decline after interference induction (Sperl and Cañal-Bruland 2020b, c). In this context, another recent study by Bruno et al. (2020) provided first evidence for reduced inhibition-related EEG activity in a Go-Nogo task following long-term limb immobilization of one week.

To gain further insights into the neuro-cognitive mechanisms and their relevance for motor skill change, the present study aimed to investigate ERP patterns associated with the physical restriction of undesired movements with regard to interference arising from an already existing, complex motor skill. Here we administered the typing paradigm with skilled touch-typists and included a rule change that prohibited one particular finger for further typing. After testing for the previously observed association between individual prepotent response inhibition abilities (measured via Stop-Signal Task) and success of interference control, we investigated the effects of a motor restriction. Specifically, we hypothesized that a motor restriction would immediately reduce the cognitive effort when implementing the rule change, and thereby diminish interference from strong prepotent response tendencies. At the behavioral level, this may be visible in a smaller performance decline in a condition with a motor restriction compared to a condition in which a rule change needs to be implemented without further assistance (hence by cognitive strategies only). Regarding ERPs, similar to Sperl et al. (2021a), first we investigated group differences in the P3 component; however, alterations in the design made it unclear whether this effect would be prominent also in the present study. Most importantly, since in the present task a prepotent response is not stopped but rather replaced by an alternative response (see also stop vs. stop-change paradigms; Boecker et al. 2013), we focus on response-locked ERPs prior to the response—a technique that is usually challenging in inhibition research. Specifically, we expected to find significant ERP differences in the pre-response interval prior to pressing a critical key depending on whether or not a motor restriction was used, considering that these differences would track the different demands to pre-response motor inhibition in these two conditions. Thereby, the present study fills a gap in the current knowledge about the neuro-cognitive effects of a short-term motor restriction to reduce interference from highly skilled motor behavior in the response-preceding interval.

## Methods

### Participants

Twenty touch-typists (15 female, mean age: 25.1 years, *SD* = 5.2, range: 17–34) contributed data to the experiment.^2^ Inclusion criteria were (a) age range 18–35 years, (b) right-handedness, (c) native speaker of German and using a German QWERTZ keyboard, (d) minimum typing speed of 30 words per minute and (e) no report of neurological or psychiatric diseases. The average typing speed was 254 characters per minute (51 words per minute, respectively). All participants regularly used the touch-typing system, typically acquired through formal training (i.e., courses at school, vocational or online training) with an average experience of 10.5 years (range: 1–21). Participants received financial reward (10 €) or course credit for participation. The experiment was approved by the ethics committee of the Faculty of Social and Behavioural Sciences of the Friedrich Schiller University Jena (reference: FSV 19/071) and conducted in line with existing measures to contain the COVID-19 pandemic, following a strict Hygiene and Infection Prevention Plan of the Friedrich Schiller University Jena to ensure all involved individuals’ safety and health.

### Materials

#### Stop-Signal Task

Since prepotent response inhibition as a subdimension of inhibition has been theorized and observed to play a particular role in the success of interference control in motor tasks (Friedman and Miyake 2004; Sperl et al. 2021a, b; Sperl and Cañal-Bruland 2020b), also in the present study, we assessed this cognitive ability prior to the main experiment. The Stop-Signal Task is a well-established tool to measure general prepotent response inhibition abilities (Logan 2015). This cognitive test involves a simple classification task with the additional requirement to stop the current response whenever a tone is presented during the response process, hence a response process which is already in process needs to be interrupted. For details, refer to the supplementary information in Online Resource 1 (Section A).

#### Typing task

##### Equipment

**Fig. 1:**
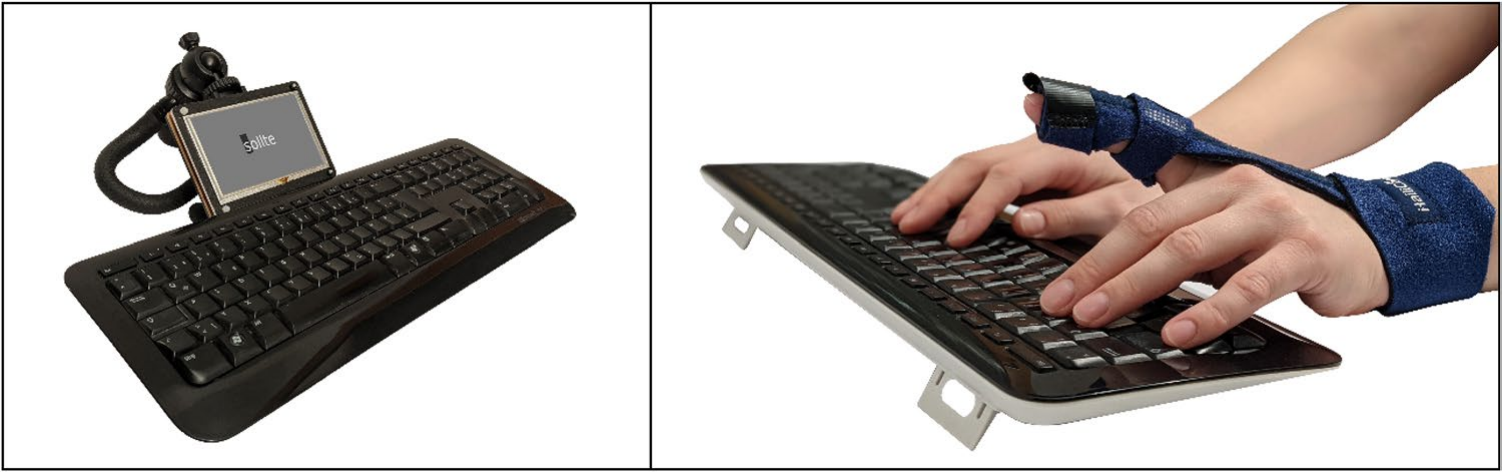
Left: typing set-up involving the external LCD monitor placed directly above the keyboard. Right: finger bandage used during Rule Change block to fixate the left index finger as motor restriction

The typing task was conducted on a standard German QWERTZ keyboard (Microsoft Wired Keyboard 400). To reduce eye movements to a minimum while allowing for visual control of hands and keyboard as in natural typing, an external 5 inch LCD monitor (Waveshare) was placed directly above the keyboard (see Fig. 1). Presenting to-be-typed stimuli in direct proximity to the keyboard minimized eye or head movements when switching attention between monitor and keyboard. A chin rest (adjustable in height and inclination) was designed to restrict head movements. Furthermore, an individually adjustable finger bandage (HailiCare) was used as motor restriction to immobilize the left index finger (see Fig. 1).

##### Word stimuli

Word stimuli were German words (length range: 3–8 characters). All words (including nouns) were presented in lower case letters, ruling out the need to use the shift key when typing.

Critical words included one of the six letters which following the touch-typing system are mapped to the left index finger (i.e., R, F, V, T, G or B). The complete set of word stimuli (240 words) fell into three classes of stimuli (80 words each): no critical letters (e.g., *sehen*), critical letter in the first position (e.g., *reden*) and critical letter in the fifth position (e.g., *dichten*). The complete stimulus list and details on stimulus selection can be found in Online Resource 1 (Section B). To compare the two conditions irrespective of any influence of stimulus difficulty on typing performance, we used the same stimuli in both Baseline and Rule Change (see also Anderson et al. 2009; Gordon et al. 1994; Parasher et al. 2001; Sperl and Cañal-Bruland 2020c). We conducted ERP analyses only for trials with critical letters in the first position (stimulus- and response-locked) since ERPs for trials with critical letters in the fifth position were likely contaminated by previous keystrokes. Behavioral data were analyzed for both positions.

##### Typing software

A script using PsychoPy 3.6 (Peirce, 2007) was programmed to present stimulus words, measure typing performance, i.e., Interkeystroke Interval (IKSI; time from one keystroke to the next one, i.e., the reaction time for every separate keystroke^3^) and errors, and send stimulus and response triggers to the EEG amplifier. Stimulus words were displayed centered in white color (font: MS Reference Sans Serif, font size: 120) on black background, always preceded by a fixation cross of 1700 ms. The current to-be-typed letter was always highlighted by a grey frame; correctly typed letters turned into green, incorrectly typed letters turned into red color. In case of an error, participants had to correct the last entry by pressing the correct key to continue (no backspace key required).

#### Questionnaire

A short questionnaire included four questions regarding touch-typing experience and typing habits.

#### EEG recording

Electrophysiological data were recorded continuously using a 32-channel EEG with BioSemi Active II system (BioSemi, Amsterdam, The Netherlands). The sampling rate was 512 Hz from DC to 155 Hz. EEG recording sites included Fz, Cz, Pz, Iz, FP1, FP2, F3, F4, C3, C4, P3, P4, O1, O2, F7, F8, T7, T8, P7, P8, F9, F10, FT9, FT10, TP9, TP10, P9, P10, PO9, PO10, I1, I2 and four additional EOG electrodes (one each above and below the right eye and one each at the outer canthi of right and left eye). Note that the BioSemi system uses a so-called “zero-Ref” system which uses two additional electrodes (CMS and DRL) instead of reference and ground electrode (see also www.biosemi.com/faq/cms&drl.htm).

### Procedure

Prior to the experiment, participants conducted a 1-min online typing test which measured typing speed. On arrival in the lab, participants provided informed consent and confirmed the absence of COVID-19 symptoms or recent risk contacts combined with a body temperature check. After procedural briefing and completing of the short questionnaire, they started with the Stop-Signal Task, then followed by the main EEG experiment (EEG cabin: IAC^TM^CT-400).

*Baseline:* first, all participants were instructed to type the 240 stimulus words (in random order) in the habitual manner as accurately and fast as possible. For familiarization, participants absolved a short practice list of nine stimuli (three of each word category), prior to this main task. After every 20 trials, participants were invited to take a short break. This task was identical for both groups.

*Rule Change:* in the subsequent block, the critical rule change was introduced to participants who now typed the same 240 words (again presented in random order) following the new rule to not use the left index finger (again, practice unit and breaks were included). Importantly, participants were randomly assigned to one of two groups at the beginning of the experiment. Whereas the *verbal instruction (VI) group* performed this task without any constraints, the *additional motor restriction (AMR) group* was provided with a critical motor restriction (see Fig. 1), that prevented movement of the left index finger. Hence, a between-subject design was applied. Any accidental rule breach in the VI group (i.e., typing with the left index finger despite of the new rule) was indicated by the experimenter by an auditory signal, reminding the participant to follow the new rule and marking this trial as invalid for data analysis.

### Data analysis

#### Behavioral data

Behavioral data were pre-processed in R Studio 1.1.419 (RStudio Team 2020) and statistically analyzed using the software JASP 0.14.0 (JASP Team 2020). The typing parameters IKSI and errors were computed for each participant. IKSI was computed based on directly correct responses only (hence, no presence of previous errors on the same letter). Errors were corrected for multiple error occurrences. This means that multiple errors on the same letter were transformed into only one error, hence, a response to one particular letter in the stimulus was counted as either correct or false. This computation avoids overweighting of multiple errors on the same key that happen ﻿e.g. when the participant does not notice an error and continues typing the subsequent letters. To examine behavioral changes in typing performance, a 2 (block: Baseline vs. Rule Change) × 2 (group: AMR vs. VI) × 2 (position: 1 vs. 5) ANOVA on errors on critical keys was conducted. Following the advice of an anonymous reviewer, for IKSI we conducted this analysis separately for each critical position (cf. Logan and Crump 2009), resulting in two 2 (block: Baseline vs. Rule Change) × 2 (group: AMR vs. VI) ANOVAs on IKSI.

In addition, correlation analyses were carried out to test for statistical associations between prepotent response inhibition abilities (reflected by SSRT from the Stop-Signal Task) and the amount of interference in the typing task (reflected by errors and IKSI for critical keystrokes in first and fifth position).

#### EEG data

EEG raw data files (.bdf files) were pre-processed administering EOG-based artifact correction (HEOG threshold: 150 µV, VEOG threshold: 250 µV) and filtering (0.3–30 Hz) in BESA Research 7.0. EEG. Data of one participant had to be excluded due to technical problems at recording, which resulted in 19 datasets (AMR group: *n* = 10; VI group: *n *= 9). The pre-processed data files were converted to .fif files and further processed in Spyder 3.8, using the MNE package (see https://mne.tools). In case of a trial including a breach of rule in the VI group, this trial was detected and excluded from further processing (on average this affected 7.2 trials per participant including 4.6% of critical trials). Then, ERPs were computed for critical trials with correct responses (i.e., trials reflecting successful inhibition). Therefore, epochs were extracted, baseline-corrected and averaged for each condition. Trials were both averaged locked to stimulus onset and to motor response (first keypress), respectively. Artifact rejection parameters were based on peak-to-peak amplitude and set to 100 µV. After artifact rejection and including only trials with correct responses (reflecting successful inhibition), 90% of the trials could be included for stimulus-locked analyses (on average over all participants, 73.4/80 total trials in Baseline, and 70.4/80 trials in Rule Change) and 65% of the trials could be included for response-locked analyses (59.4/80 trials in Baseline, 43.8/80 trials in Rule Change). For stimulus-locked analyses (interval: − 200 to 1500 ms; baseline: − 200 to 0 ms) mean amplitude was calculated in a time window of 300 to 500 ms encompassing the P3 component. A 2 (block: Baseline vs. Rule Change) × 2 (group: VI vs. AMR) × 3 (Anteriority: frontal vs. central vs. parietal) × 3 (Laterality: left vs. middle vs. right) ANOVA was conducted to check for differences in mean amplitude. Regarding the response-locked analyses (interval:  − 600 to 100 ms; baseline: 0 to 100 ms^4^), the pre-response interval was split into three consecutive and equidistant subintervals of 200 ms each for which mean amplitudes were computed. Then, the change of this mean amplitude was calculated via difference scores (for each interval), always subtracting the Baseline from the Rule Change condition. A 2 (group: VI vs. AMR) × 3 (time window: − 600 to − 400 ms vs. − 400 to − 200 ms vs. − 200 to 0 ms) × 3 (Anteriority: frontal vs. central vs. parietal) × 3 (Laterality: left vs. middle vs. right) was conducted to test for differences in mean amplitude change across groups, time windows and regions, and post-hoc t-tests (corrected for multiple tests) were performed to follow-up significant findings.

Furthermore, polynomial contrasts across the three pre-response intervals were computed within each group, to test for linear and quadratic (curvilinear) trends. They were conducted for both, the difference score as well as for Baseline and Rule Change values separately. These analyses allowed us to detect and statistically manifest group differences (both quantitative and qualitative) in trends over time in the brain activity course across the three pre-response time windows (cf. Field 2013).

Statistical analyses were conducted in Spyder 3.8 and JASP 0.14.0. Polynomial contrast analyses were carried out in IBM SPSS Statistics 27 (IBM Corp. 2020). The *α*-level for all statistical tests was set to 0.05. In case of violation of the sphericity assumption, Huynh–Feldt (Huynh and Feldt 1976) corrected *p* values were used and *ε* values reported.

## Results

### Behavioral data

#### Typing performance

**Fig. 2:**
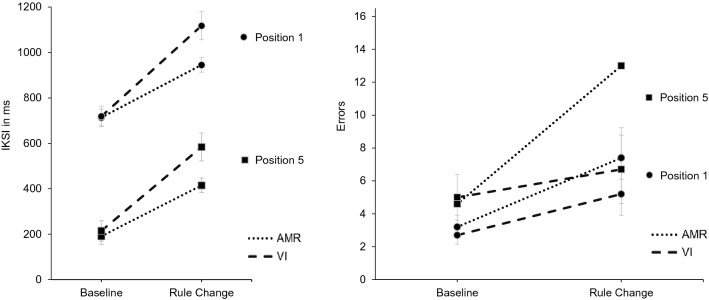
IKSI (left) and errors (right) for critical keys for each block (Baseline vs. Rule Change; *x*-axis), group (AMR vs. VI; dotted vs. dashed line) and position (first vs. fifth; circled vs. squared markers). Error bars indicate standard errors. Groups: *AMR* additional motor restriction, *VI* verbal instruction

In typing performance, we observed the expected main effects of block and position, in the absence of clear differences between groups (Fig. 2).

Specifically, the 2 (block: Baseline vs. Rule Change) × 2 (group: AMR vs. VI) ANOVA on IKSI for critical keys in the first position (correct responses only) revealed a main effect for block [*F*(1, 18) = 49.28, *p* < 0.001, *η*_*p*_^2^ = 0.73], which was also present in the analogous ANOVA on IKSI for critical keys in the fifth position [*F*(1, 18) = 52.14, *p* < 0.001, *η*_*p*_^2^ = 0.74]. On average, IKSI increased by 232 ms (*SE* = 6 ms) in the AMR group and by 399 ms (*SE* = 7 ms) in the VI group for first position keystrokes. For fifth letter keystrokes, IKSI increased by 224 ms (*SE* = 6 ms) in the AMR group and by 369 ms (*SE* = 6 ms) in the VI group.

The ANOVA for errors on critical keys also revealed main effects for block [F(1, 18) = 8.09, *p* = 0.011, *η*_*p*_^2^ = 0.31] and position [F(1, 18) = 10.30, *p* = 0.005, *η*_*p*_^2^ = 0.36]. On average, participants of the AMR group made 4.2 more errors (*SE* = 1.8) in the Rule Change compared to the Baseline condition; in the VI group, errors increased by 2.5 (*SE* = 1.4). Regarding critical letters in the fifth position, the increase in errors was 8.4 (*SE* = 3.3) in the AMR group, and 1.7 in the VI group (*SE* = 2.3). Note that trends towards block*group interactions for both positions in IKSI was observed [position 1: *F*(1, 18) = 3.49, *p* = 0.078, *η*_*p*_^2^ = 0.16; Position 5: *F*(1, 18) = 3.14, *p* = 0.093, *η*_*p*_^2^ = 0.15] which, however, might reflect a nonreliable degree of speed-accuracy tradeoff (Fig. 2). No other effects than the reported effects were significant (for full details, see Online Resource 1, Section C).

#### Stop-Signal Task

Mean SSRT in this sample was 240 ms (SD = 35 ms). There was neither a significant correlation between SSRT and IKSI (*r* =  − 0.390, *p *= 0.110) nor between SSRT and errors (*r* = 0.047, *p *= 0.854), nor were there any significant correlations when calculated separately per group (all *p*s > 0.05).

### EEG data

#### Stimulus-locked data

The 2 (block: Baseline vs. Rule Change) × 2 (group: VI vs. AMR) × 3 (anteriority: frontal vs. central vs. parietal) × 3 (laterality: left vs. middle vs. right) on mean amplitude in the time window from 300 to 500 ms revealed no effects or interactions involving group or experimental factors, and only yielded significant results involving the topographical factors anteriority and laterality (for complete statistics, see Online Resource 1, Section D, Table 3; and waveshapes, see Appendix, Fig. 6).

#### Response-locked data

**Fig. 3:**
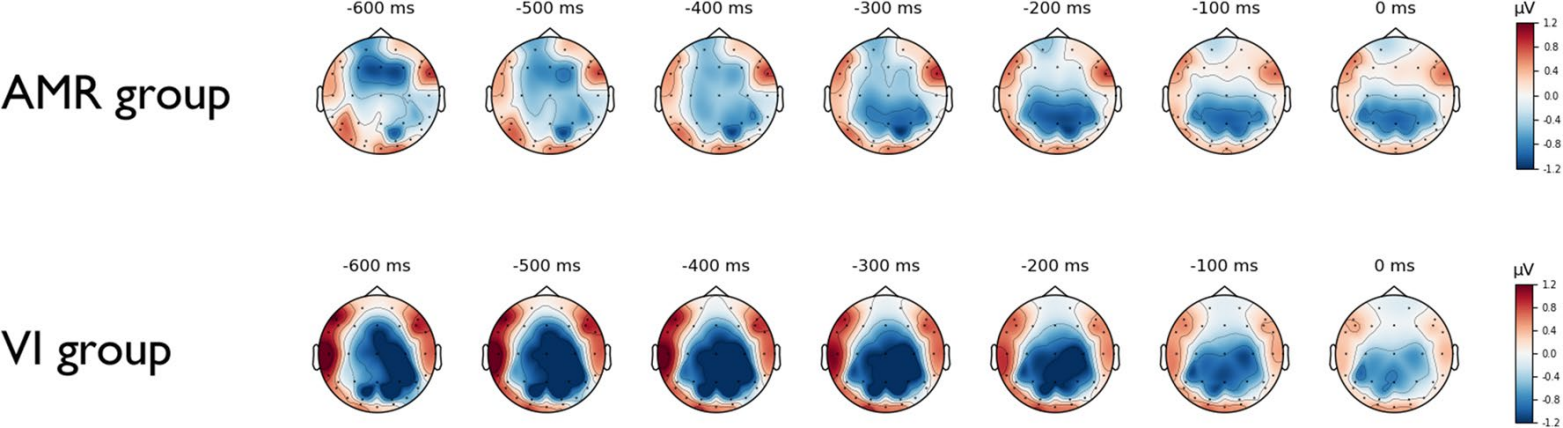
Scalp Maps depicting the course of the difference wave (Rule Change vs. Baseline) in the pre-response interval for correct keypresses for critical keys in first position for each group. *AMR* additional motor restriction, *VI* verbal instruction

**Fig. 4:**
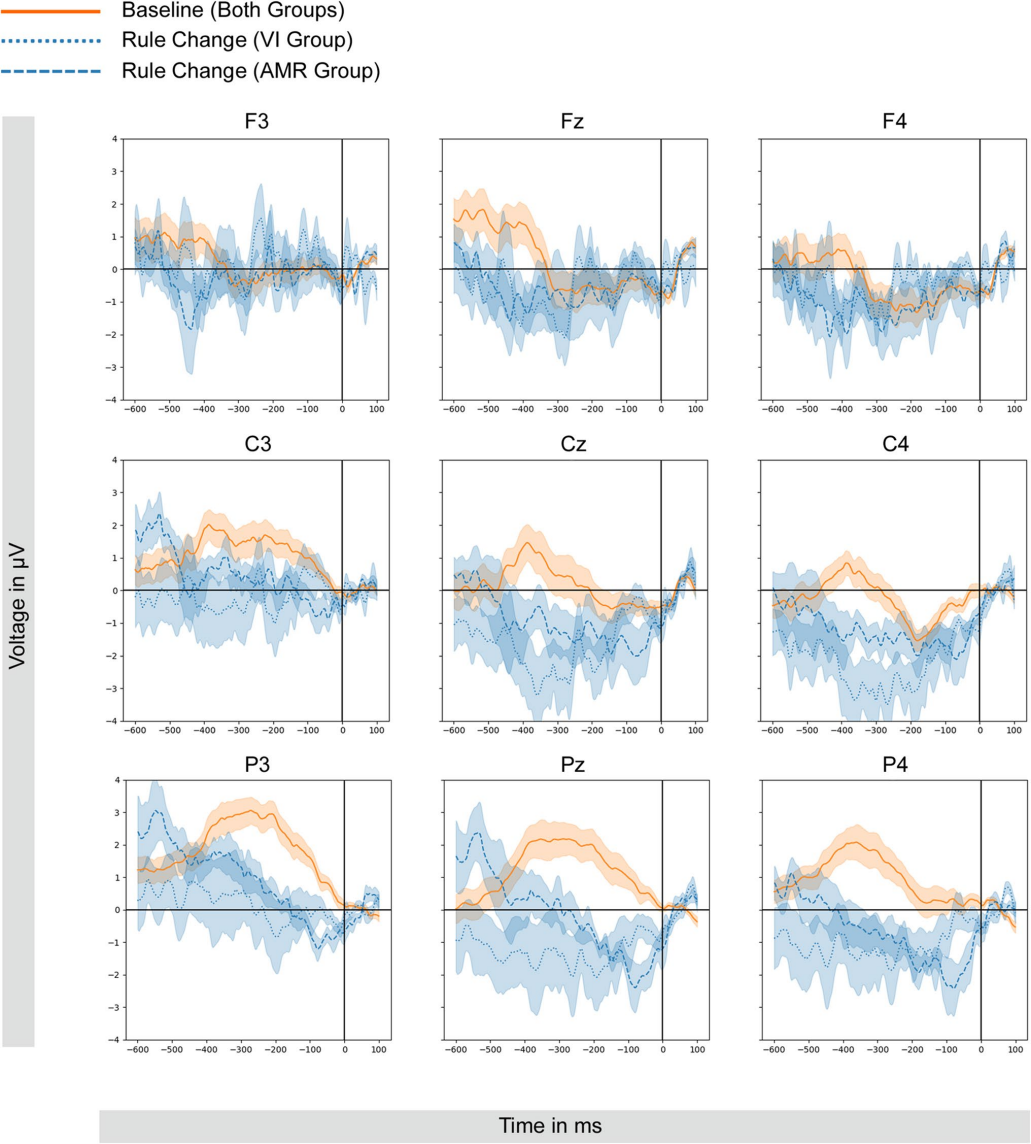
Response-locked ERPs (− 600 ms to 100 ms) for correct keystrokes of critical letter in first position. *Note:* For the sake of clarity, the orange line depicts the baseline course collapsed across both groups (since both groups experienced exactly the same baseline task and showed comparable baseline ERP; also cf. Figure 5, bottom). The dotted vs. dashed blue traces depict the rule change condition separately for each group. Transparent ribbons indicate standard errors

Response-locked analyses were conducted for critical letters in first position. Data is illustrated in scalp maps (Fig. 3) and ERP plots (Fig. 4).

**Fig. 5:**
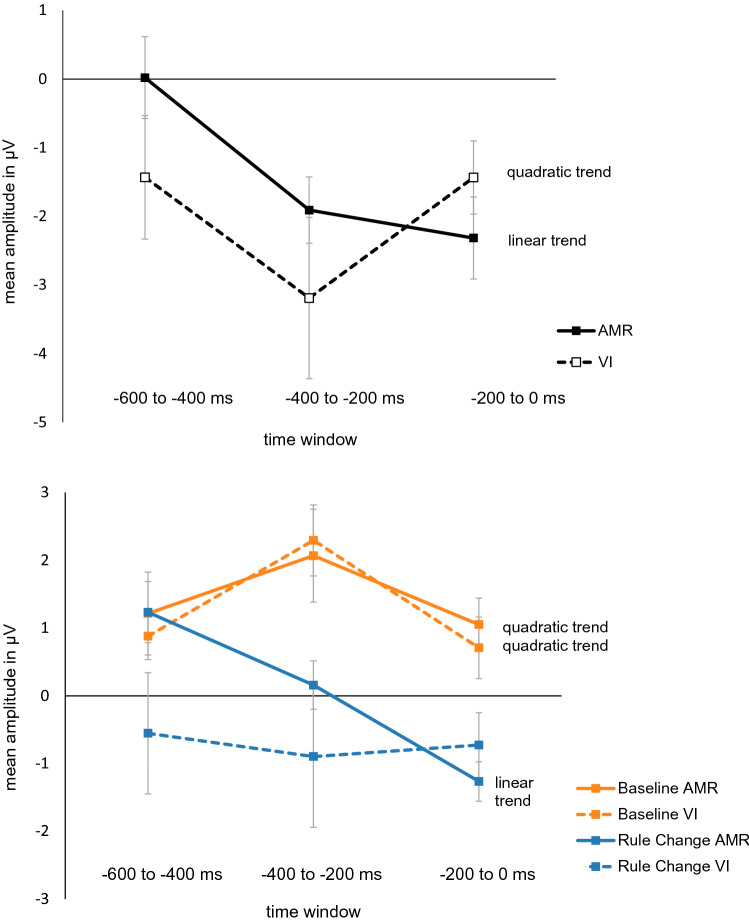
Top: Block difference in mean amplitude (Rule Change vs. Baseline) across groups and time windows for parietal sites (averaged across P3, Pz and P4). Bottom: blockwise course of mean amplitude across time windows for each group for parietal sites (averaged across P3, Pz and P4). Error bars indicate standard errors. For statistics of the depicted type of trend refer to Table 2

**Fig. 6:**
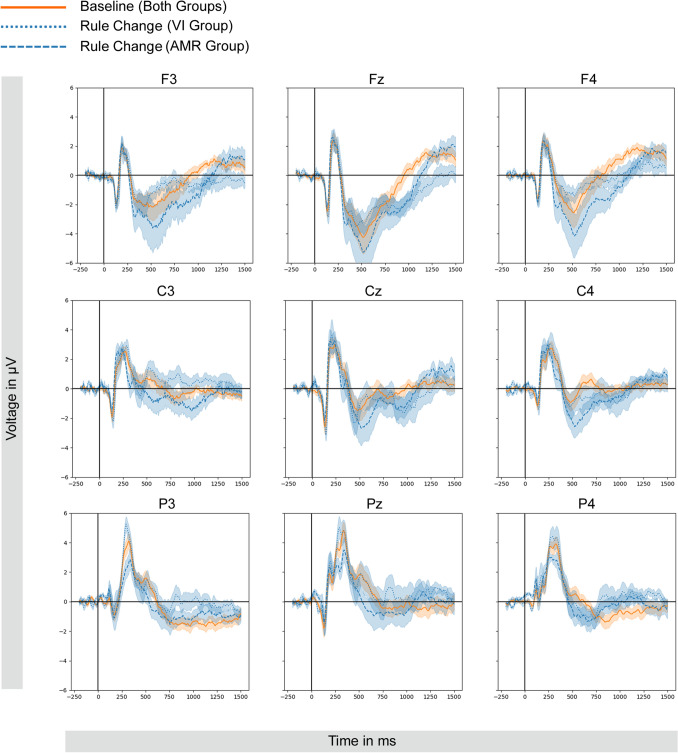
Stimulus-locked ERPs (− 200 ms to 1500 ms) for correct keystrokes of critical letter in first position. For the sake of clarity, the orange line depicts the baseline course collapsed across both groups (since both groups experienced exactly the same baseline task and showed comparable baseline ERP). The dotted vs. dashed blue traces depict the rule change condition separately for each group. Transparent ribbons indicate standard errors.

The 2 (group: VI vs. AMR) × 3 (time window: − 600 to − 400 ms vs. − 400 to − 200 ms vs.  − 200 to 0 ms) × 3 (anteriority: frontal vs. central vs. parietal) × 3 (laterality: left vs. middle vs. right) ANOVA (cf. Table 1) revealed a significant time window*anteriority*group interaction [*F*(4, 68) = 3.369, *p *= 0.038, *η*_*p*_^2^ = 0.165, *ε* = 0.586], which appeared to reflect a time window*group interaction at parietal sites in particular (Figs. 5 and 6). Separate ANOVAs for the three levels of anteriority revealed that the time window*group interaction was significant at parietal sites only [*F*(2, 34) = 3.793, *p *= 0.033, *η*_*p*_^2^ = 0.182, *ε* = 1.000], but not at frontal [*F*(2, 34) = 1.476, *p *= 0.243, *η*_*p*_^2^ = 0.080, *ε* = 0.838] or central sites [*F*(2, 34) = 1.223, *p *= 0.307, *η*_*p*_^2^ = 0.067, *ε* = 0.908]. Post-hoc tests for the parietal sites revealed a significant change to more negative amplitude from the earliest to the latest interval in the AMR group [*t*(9) = 3.596, *p *= 0.015] only (Figs. 5 and 6). No other comparisons between time windows or groups were significant (all *p*s ≥ 0.07).

**Table 1:** Main effects and interaction coefficients of the 2 (group: VI vs. AMR) × 3 (time window: − 600 to − 400 ms vs. − 400 to − 200 ms vs. − 200 to 0 ms) × 3 (anteriority: frontal vs. central vs. parietal) × 3 (laterality: left vs. middle vs. right) on mean amplitudes

Effect	*df*	*F*	*p*	*η*_*p*_^2^	*ε*
Time window	2, 34	2.418	.104	0.125	.939
Group	1, 17	0.706	.412	0.040	–
Anteriority	2, 34	3.239	.066	0.160	.775
Laterality	2, 34	2.321	.135	0.120	.668
Time window*group	2, 34	1.243	.301	0.068	.939
Anteriority*group	2, 34	0.806	.429	0.045	.775
Laterality*group	2, 34	1.210	.299	0.066	.668
Time window*anteriority	4, 68	8.105	< .001***	0.323	.586
Time window*laterality	4, 68	5.057	.001	0.229	.865
Anteriority*Laterality	4, 68	0.734	.572	0.041	.886
Time window*anteriority*group	4, 68	3.369	.038*	0.165	.586
Time window*laterality*group	4, 68	0.929	.452	0.052	.886
Anteriority*laterality*group	4, 68	0.929	.452	0.052	.886
Time window*anteriority*laterality	8, 136	4.887	< .001***	0.223	.748
Time window*anteriority* laterality*group	8, 136	0.869	.520	0.049	.748

*p < .05

***p < .001

Figure 5 illustrates the course of the block difference in parietal amplitude (P3, Pz and P4) across the three time window for each, and mean amplitudes per group for Baseline and Rule Change block separately. Results of polynomial contrast analyses confirmed the visual impression (Fig. 5; for full statistics see Table 2). In short, whereas rule change elicited a quadratic trend (u-shaped) in the difference wave for the VI group [*F*(1, 8) = 6.488*, p *= 0.034, *η*^2^ = 0.448], a linear trend was seen in the AMR group [*F*(1, 9) = 13.234*, p *= 0.005, *η*^2^ = 0.595]. Figure 5 (bottom) confirms that both groups show a comparable pattern of ERPs at Baseline in which they exhibit an inverted u-shaped quadratic trend [AMR: *F*(1, 9) = 7.396*, p *= 0.024, *η*^2^ = 0.451, VI: *F*(1, 8) = 17.272,* p *= 0.003, *η*^2^ =  0.683] with a maximum positive voltage around − 400 to − 200 ms. In the Rule Change, however, the AMR group’s voltage sets in at more or less the same mean amplitude as in the Baseline and is then continuously negative-going [emphasized by the linear trend; *F*(1, 9)  = 35.694*, p *< 0.001, *η*^2^ =  0.799)] The VI group, however, already in the earliest pre-response-interval (− 600 to − 400 ms), reveals a much more negative mean amplitude which remains constant until the execution of the keypress [no linear trend: *F*(1, 9) = 0.102*, p* = 0.757, *η*^2^ = 0.013].

**Table 2:** Coefficients of the polynomial contrasts for factor time window for Baseline, Rule Change and difference score for both groups

Polynomial contrast	AMR			VI			
	*F (1, 9)*	*p*	*η*^2^	*F (1, 8)*		*p*	*η*^2^
Baseline							
Linear	0.070	.797	0.008	0.136		.721	0.017
Quadratic	7.396	**.024**	0.451	17.272		**.003**	0.683
Rule Change							
Linear	35.694	** < .001**	0.799	0.102		.757	0.013
Quadratic	0.221	.650	0.024	0.307		.595	0.037
Difference Score							
Linear	13.234	**.005**	0.595	< 0.001		.997	< 0.001
Quadratic	3.028	.116	0.252	6.488		.**034**	0.448

Significant *p*-values are printed in bold

## Discussion

In this study, we investigated brain correlates of successful interference control in a complex motor skill, either with or without motor restriction. Therefore, skilled touch-typists were confronted with a rule change that required to suppress certain finger movements during typing and replace those by alternative actions, posing high demands on the inhibition of strong, automatized response tendencies. Whereas one group received a motor restriction which physically precluded the to-be-suppressed automatic action, a second group had to implement the new rule without further assistance (hence by cognitive strategies only).

First, in line with previous findings (Sperl and Cañal-Bruland, 2020b, c), behavioral results revealed a large proactive interference effect after introduction of the rule change, visible in substantial increases in both IKSI and errors from Baseline to Rule Change (see Fig. 2). While these basic effects were substantial and clear, visual inspection further indicated a potential differential speed-accuracy tradeoff across groups, suggesting different adaptations to the rule change. Specifically, in the AMR group, rule changes caused marginally smaller costs in IKSI, which were accompanied by numerically larger costs in accuracy, compared to the VI group. While these observations could be plausible and potentially interesting, these group differences were not statistically significant. We, therefore, refrain from further speculation, as high-powered experiments seem required to assess the reliability of these observations. The same appears to apply for the non-significant results regarding the Stop-Signal Task. While previous studies applying a similar typing task design revealed significant correlations between this response inhibition task and typing performance (Sperl and Cañal-Bruland, 2020b; Sperl et al. 2021b), no significant correlations were observed in the present study. This might either be due to methodological differences in the task (continuous text vs. single words as stimuli) or to limited power for these tests which were not the central focus of the present study (see also power analysis in Methods section).

The stimulus-locked analyses revealed neither block nor group to modulate the response inhibition-related P3 component. Relative to our previous study (Sperl et al. 2021a), where considerably large P3 effects were present, we assume that the absence of this component might relate to differences in the rule change manipulation. In the present task, the target key remained identical but had to be pressed by a different effector (which was different in the letter switch paradigm; Sperl et al. 2021a). While in line with the previous study participants still had to inhibit the tendency to use the actual finger, here, the target location of the to-be-typed letter remained the same. Hence, while still requiring the inhibition of a prepotent action tendency, the present task may call for a different technique to achieve the same target, which in turn seems to have important influences on the process of response inhibition and the associated ERP patterns. In fact, previous research has shown how subsequent actions after the original inhibition process appear to crucially modulate known ERP effects related to inhibition (cf. Krämer et al. 2011). Thus, the type of the rule change constraint might be crucial in these experimental studies (for a related discussion, see also Sperl et al. 2021b). While not being the focus of the present study, future research is required to gain a more thorough understanding of how the nature of each interference task acts on processes of inhibition and its associated ERPs.

Most importantly, response-locked ERPs revealed significant differences in the pre-response interval of successfully pressed critical keys. While both groups showed higher levels of movement-preceding negativity in the Rule Change block (compared to Baseline), brain waves revealed a different time course of this negativity, indicating critical disparities in the preparatory period depending on the group (see Fig. 4), which appeared to be most prominent at parietal sites. Specifically, the VI group revealed a more or less constant negativity prior to the keystroke. In contrast, the AMR group’s ERP course started at a level that was highly comparable to baseline activity (around 600 ms prior to the keystroke) with a continuously increasing negativity reaching the amplitude of the VI group in the interval right before the keystroke (− 200 to 0 ms; see Fig. 5). While the observed movement-preceding negativity is present in both groups, we assume that the different spatiotemporal patterns of preparatory EEG activity reflect the specific task demands in the present study which require to prepare for a novel motor act while inhibiting a familiar motor response. In this context, the present results may suggest that the VI group engages inhibitory and motor preparation processes earlier and with a sustained time course which may reflect higher cognitive control demands to motor preparation in the absence of a motor restraint. In contrast, benefits from motor restriction in the AMR group may involve reduced cognitive control demands to motor preparation, such that these preparatory processes can be invoked later in time, with a maximum of negativity only right before the critical keystroke. Possibly, less motor preparation is required when the option to move the critical finger is physically withdrawn, and inhibitory processes are therefore demanded to a smaller extent.

Indeed, similar effects were observed for inhibition-related EEG in phantom limb research, where less activity is observed for static compared to moving phantom limbs in response inhibition tasks (Bruno et al. 2019). Moreover, a similar movement-preceding negativity has been typically reported in the context of Bereitschaftspotential (Deecke et al. 1969; Jahanshahi and Hallett^2003^). Although often associated with a (fronto-)central maximum, this phenomenon also has been reported to occur maximally in the centro-parietal areas (Shibasaki and Hallett 2006) and was observed to occur/start especially at parietal regions when so-called *praxis movements* are subject of the experimental task, i.e., movements that are usually employed in daily life (Shibasaki and Hallett 2006; Wheaton et al. 2005). This indeed corresponds to our experimental task addressing the motor skill of typing. While this potential is typically elicited by spontaneous and self-paced movements (Schurger et al. 2021), the larger negativity in the VI group in our task accords well with several factors that had been observed to increase the magnitude of the Bereitschaftspotential, such as perceived effort, complexity and discreteness (for an overview, see Shibasaki and Hallett 2006). Also, speed of movement appears to modulate this potential as it has been argued that it occurs closer to the response the faster an action is executed (Shibasaki and Hallett 2006), which, in fact, seems to accord well with our behavioral data which indicate that the AMR group tends to show shorter reaction times after the rule change than the VI group.

To summarize, the motor restriction indeed appears to modulate changes in preparatory brain potentials, probably in terms of facilitated response inhibition. Notably, building upon recent evidence by Bruno et al. (2020) who observed that one week of limb immobilization significantly modulated physiological responses (assessed via EEG and TMS), our findings suggest that differences in the inhibitory preparatory phase following motor restrictions are, in fact, already visible at a short-term level without involving long-term plasticity (i.e., here, within one task block of approximately 15 min).

Important to note, in this motor skill change task, dominant action components had to be replaced by alternative actions, rather than stopping them at all. Hence, while certainly containing important components of response inhibition, it should be kept in mind that the pre-response interval may likely contain also other response-related mechanisms, such as the selection of the alternative response (and hence contain both inhibitory and preparatory components). In fact, the present task reflects a stop-change rather than a pure stop-task (for an overview, see Boecker et al. 2013). Administering a pure stop-task, however, would not have enabled to investigate this topic in the light of motor skill change as it occurs in real life (where actions are usually replaced by new ones rather than omitted at all), while the present paradigm additionally offered the unique possibility to gain a sophisticated look on response-locked ERPs associated with inhibition and interference control in motor tasks.

Further limitations of the current design might reside in the fact that participants were free to choose how to implement the new rule, i.e. how to replace the to-be-avoided finger after the rule change. Providing participants with specific instructions which finger to use might allow to also analyze lateralized effects and gain further insight into motor preparation processes, while, however, reducing the ecological validity of the task at the same time. Moreover, future studies may investigate whether the strategy of replacement also depends on the presence or absence of a motor restriction and further on the specific location of the target letters (i.e., R, F, V vs. T, G, B). Furthermore, previous studies showed that while typing a key with the target hand, inhibition of the non-relevant hand seems to take place and that this is electrophysiologically reflected by ipsilateral positivity in the pre-response interval (interpreted as inhibition) over motor areas (C3/C4), while the contralateral side typically shows a pre-response negativity (Pinet et al. 2015; Vidal et al. 2003). In fact, our data seem to be in line with this pattern, at least for the Baseline condition, where the first keystrokes were necessarily typed by the left index finger (i.e., pre-response positivity at C3 (ipsilateral), hence inhibition of the right hand, and pre-response negativity at C4 (contralateral); see Fig. 4).

Building upon this thought, in typing research, also analyses of LRPs turned out to provide a sophisticated measure to understand processes of response preparation and inhibition (Pinet et al. 2015; Scaltritti et al. 2018). Also for the current research question, LRP analyses might offer a future approach as it additionally allows to analyze lateralized effects which might be, for instance, interesting with regard to the question which alternative finger had been used when dealing with the rule change. Moreover, future studies might investigate early stimulus- or response-locked activation (in the time interval between stimulus onset and first keypress) also for critical letters located later in the word, thereby generating further knowledge about the scope of inhibition (cf. studies on early vs. late deviant keys by Logan et al. 2011; Scaltritti et al. 2018). In fact, delays on adjacent keystrokes (i.e., before and after a critical keystrokes) suggest that interference effects are not necessarily localized to critical keystrokes only (see, e.g., Yamaguchi and Logan 2014). Comparable delays in typing times (as well as neural effects) already before the critical letter could also be observed, for example, in a recent study by Palmis et al. (2019) which investigated the effects of orthographic errors on motor behavior in handwriting (even though comparability with the present study is certainly limited due to conceptual and methodological differences).

Finally, it should always be kept in mind that variations in trial numbers as well as baseline contamination as a common issue for response-locked analyses are known to easily modulate amplitudes and courses of components (see e.g., Luck 2014). Baseline correction can be done in various ways and there is no absolute gold standard for response-locked analyses (for alternatives and discussions, see e.g. Alday 2019; del Río et al. 2018; Luck 2014). Also, when conducting ERP research the number of statistical analyses and ANOVA factors need to be carefully considered (Luck and Gaspelin, 2017). In addition, future studies with larger sample sizes might also allow to detect correlational relationships between behavioral and electrophysiological data.

Summing up, together with the study by Bruno et al. (2020), this is one of the first studies that combined the idea of motor restriction as a potential inhibition support with EEG. In fact, this field of research bears a high potential to understand the neural correlates associated with successful interference control in motor tasks. However, while certainly providing valuable insights into the brain correlates of motor restriction, more research is required to gain a deeper understanding of the cognitive processes that underlie the presented different ERP courses and disentangle inhibitory and preparatory processes in the pre-response period. Future studies might further combine behavioral assessments with different neuroscientific methods such as EEG, TMS, fMRI, but also EMG (cf. Bruno et al. 2020; Facchini et al. 2002; Garbarini et al. 2019; Liepelt et al. 2009) to gain a deeper understanding about the brain regions involved in these processes, but also to be able to investigate the behavioral effects (and the interplay between both) which might be relevant for practical applications.

## Conclusion

To conclude, the present findings indicate that a motor restriction modulates preparatory brain activity which was visible already at a short-term level. Whether these changes indeed represent facilitation of inhibitory processes in motor tasks remains to be determined. Future research is needed to investigate the neuro-cognitive processes necessary to deal with interference from pre-existing, procedural skills which might be relevant for various types of motor skill change, may it be in the context of sports, medicine or daily life activities.

### Supplementary Information

Below is the link to the electronic supplementary material.

Supplementary file1 (PDF 252 KB)

## Data Availability

The data generated and analyzed during the current study are available from the corresponding author on reasonable request.

## Footnotes

1 Note that response inhibition is also often reported to be reflected by the N2 component or an N2/P3 complex respectively (for a review, see e.g., Huster et al. 2013). However, several studies have shown that this is different for so-called stop-change tasks (Boecker et al. 2013). Specifically, it has been shown that, whenever a response does not only need to be stopped, but also replaced by an alternative response, this is typically accompanied by enhanced P3 components in the absence of any N2 effects (see Krämer et al, 2011; Xie et al. 2017), which was indeed also found in the mentioned study by Sperl et al. (2021a).

2 An a priori power analysis was conducted with G-Power 3.1.9.2. A repeated measure ANOVA with within-between interaction was used as main effect of interest. Using an alpha of .05, a power of .80, an effect size F of .25 and non-sphericity correction ε = 1, this analysis revealed a minimum required sample size of *n* = 18. The correlation among repeated measures was computed based on previous data of a similar experimental design by Sperl et al. (2021a) (*r* = .758).

3 For the first keystroke, IKSI refers to the time interval from stimulus onset to the first keystroke (in other typing studies, it is also referred to as “response time” or “reaction time”; e.g., Snyder & Logan, 2013; Yamaguchi et al., 2013).

4 Note that selection of an adequate baseline period for response-locked analyses can be a critical issue, and that there are various alternatives of how to perform adequate baseline correction. Since, in the current study, we were interested in the *pre*-response interval, we refrained from choosing a pre-response baseline period, which is often chosen in response-locked analyses. Specifically, we considered that in the current paradigm, any interval right before the pre-response time window of interest might be contaminated by previous events, such as the stimulus onset (depending on response speed). Moreover, we decided against a fixed pre-stimulus baseline period because response times largely vary across trials and conditions and this time window might be rather distant from the time window of interest, which can be problematic with regard to slow potential drifts. Therefore, we chose a considerably short *post*-response baseline period which is assumed to represent an average offset of a particular trial, and which is rather uncontaminated by other events, while at the same time being close to the time window of interest (see also discussion section for additional literature). Despite these considerations which guided our choice here, we also performed additional analyses with a baseline period from -600 ms to 100 ms upon an anonymous reviewer suggestion. These alternative analyses revealed largely comparable results to the ones we report here.
