# Supplementary material for: Effects of motor restrictions on preparatory brain activity
Source: Exp Brain Res. 2021 Aug 25;239(11):3189–203. doi: 10.1007/s00221-021-06190-w (PMC8386343; doi:10.1007/s00221-021-06190-w)
Supplement: Supplementary file 1 — Supplementary file1 (PDF 252 KB) [file 221_2021_6190_MOESM1_ESM.pdf]

Online Resource 1 for Manuscript:

Effects of motor restrictions on preparatory brain activity in skilled touch-typists

Sperl, L.[1 2], Ruttloff, J. M.[2], Ambrus, G. G.[3], Kaufmann, J. M.[2], Cañal-Bruland, R.[1] & Schweinberger, S. R. [2]

[1] Department for the Psychology of Human Movement and Sport, Institute of Sports Science, Faculty of Social and Behavioural Sciences, Friedrich Schiller University Jena

[2] Department of General Psychology and Cognitive Neuroscience, Institute of Psychology, Faculty of Social and Behavioural Sciences, Friedrich Schiller University Jena

[3] Department of Biological Psychology and Cognitive Neurosciences, Institute of Psychology, Faculty of Social and Behavioural Sciences, Friedrich Schiller University Jena

Correspondence concerning this article should be addressed to Laura Sperl, Department of General Psychology and Cognitive Neuroscience, Institute of Psychology, Faculty of Social and Behavioural Sciences, Friedrich Schiller University Jena, Am Steiger 3, 07743 Jena, Germany; E-mail: laura.sperl@uni-jena.de

Supplementary Information

Section A – Detailed description of the Stop-Signal Task

The Stop-Signal Task is a well-established tool to measure response inhibition (Logan, 2015). We used the STOP-IT software provided by Verbruggen et al. (2008) which is accessible on the Open Science Framework platform (https://osf.io/wuhpv). Within this task, participants perform a simple decision task where they are instructed to press a respective button (right or left) when one of two geometric forms is displayed (circle vs. square). Participants continue this classification task until during a trial an auditory signal tone is played. In this case, participants are instructed to immediately interrupt their current response and refrain from pressing the response button in this trial. Trials without auditory signal are performed as usual, i.e. classifying the geometric form. In the present study, participants first absolved a practice block of 1x32 trials, followed by the main task which included 3 blocks of 64 trials with a short break after each block. Importantly, the Stop-Signal Task is adaptive, i.e. the time between the stimulus and the stop signal (stop signal delay) varies depending on the participant's previous test performance. Specifically, the stop signal delay always starts at a default value of 250 ms and increases by 50 ms after a successful inhibition trial and decreases by 50 ms after each unsuccessful trial. This adaptive testing results in a reliable estimation of the individual stop signal reaction time (SSRT), which allows to estimate the covert latency of the stop process (Verbruggen et al., 2008). Here, SSRT was estimated via the integration method and lower values represent better response inhibition (for a detailed description, see Verbruggen et al., 2013).

Section B – Stimulus materials

Table 1

List of the word stimuli used in the typing task

critical letter [R]/[F]/[V]/[T]/[G]/[B]					
position 1		position 5		none	
glück	vanille	ändern	ansetzen	sehen	speise
radio	glocke	eimer	hausbau	achse	lied
reisen	vase	anders	eilig	heim	musik
radius	gondel	anker	eckig	kind	lampe
glühen	bahn	dauernd	essig	klein	kleid
gleis	bach	admiral	anzüge	allein	hose
rahmen	binden	allerlei	anlage	dusche	schuh
rennen	blume	anhören	ansage	mund	ozean
gedanke	video	akkord	einige	eins	edel
rand	biene	anspruch	adlige	wunsch	nadel
ranke	viel	dampfen	anheben	wind	eiche
glas	villa	knopf	ausüben	dach	eilen
raps	vision	knauf	daneben	dunkel	einmal
galopp	visuell	schafe	anpöbeln	hell	insel
rascheln	buch	klopfen	wandbild	seele	packen
rassel	vize	dumpf	acker	loch	paddeln
gemälde	vokal	umzüge	üppig	kunde	palme
real	volk	kämpfen	chlor	kommen	schluss
rechnen	voll	knüpfen	licht	sein	pedal
reden	volumen	pazifik	adler	sonne	polizei
geschick	boden	handvoll	knöpfe	mond	jacke
fackel	tasche	penibel	sümpfe	wolke	jemand
baum	tadel	kochbuch	zwölf	ausnahme	jeweils
fahne	tal	mühevoll	symptome	zusammen	juni
bühne	tank	sinnvoll	möchten	endlich	malen
fallen	tanne	qualvoll	kajüte	ende	mai
bande	tanzen	klauben	yacht	kochen	mahl
familie	thema	schubsen	dämpfen	anziehen	medizin
finden	tauchen	anhaben	empören	zoll	mechanik
veilchen	ton	sklave	klang	name	mehl
tendenz	teilen	damit	zypern	niemand	medaille
visum	vene	demut	likör	pause	medien
feld	tempo	einst	hypothek	danken	lexikon
fuchs	gala	ansatz	wahlfach	wollen	melodie
fundus	ganzes	anmuten	kühlt	wanne	mensch
funke	gelenk	dichte	spült	see	modell
flicken	backen	akustik	kippt	deckel	nase
filiale	baden	apostel	huldvoll	hund	denken
film	balken	asiate	zenit	linse	leinwand
finale	besuchen	dichten	könig	sammeln	kolonne

Note: Stimulus words included all letters of the German alphabet apart from the letter ß which is located in the row of special signs on the QWERTZ keyboard. We opted for the present categorization based on two intentions. First, placing the critical letters (which will later be affected by the rule change) in the first position enabled us to measure ERPs which are uncontaminated by previous keystrokes, and allowed for both stimulus- and response-locked analyses (cf. Pinet et al., 2015; cf. Pinet et al., 2019; Scaltritti et al., 2018). At the same time, neither the presence nor the position of the critical letter should be predictable as this would enormously reduce proactive interference. Moreover, interference was expected to be particularly high when the critical letter is located later in the word, because by then, typing flow has already built up triggering prepotent responses (cf. Sperl et al., in revision). Therefore, also words with critical letters on the fifth position and words without any critical letters were included in the stimulus set.

Section C – Behavioral Data

Table 2

Results of the 2 (block: Baseline vs. Rule Change) x 2 (group: AMR vs. VI) ANOVA on IKSI for critical keys (Position 1), the 2 (block: Baseline vs. Rule Change) x 2 (group: AMR vs. VI) ANOVA on IKSI for critical keys (Position 5) and the 2 (block: Baseline vs. Rule Change) x 2 (group: AMR vs. VI) x 2 (position: 1 vs. 5) ANOVA on errors for critical keys.

**Effect**	***df***	***F***	***p***		**η_p^2**
		**IKSI (Position 1)**			
Block	1, 18	49.28	<.001	***	0.73
Group	1, 18	3.76	.068		0.17
Block*Group	1, 18	3.49	.078		0.16
		**IKSI (Position 5)**			
Block	1, 18	52.14	<.001	***	0.74
Group	1, 18	4.24	.054		0.19
Block*Group	1, 18	3.14	.093		0.15
		**Errors**			
Block	1, 18	8.09	.011	*	0.31
Group	1, 18	2.03	.171		0.10
Position	1, 18	10.30	.005	**	0.36
Block*Group	1, 18	2.02	.172		0.10
Position*Group	1, 18	0.90	.354		0.05
Position*Block	1, 18	1.39	.255		0.07
Block*Position*Group	1, 18	2.99	.101		0.14

Note: * $p < .05$, ** $p < .01$, *** $p < .001$.

Section D – Stimulus-locked ERPs

Table 3

Main effects and interaction coefficients of the 2 (block: Baseline vs. Rule Change) x 2 (group: VI vs. AMR) x 3 (Anteriority: frontal vs. central vs. parietal) x 3 (Laterality: left vs. middle vs. right) on mean amplitude in the time window from 300 to 500 ms

**Effect**	***df***	***F***	***p***		**η_p^2**	**ε**
Block	1, 17	1.616	.221		0.087	-
Group	1, 17	1.066	.316		0.059	-
Anteriority	2, 34	27.154	< .001	***	0.615	.643
Laterality	2, 34	2.457	.101		0.126	1.000
Block*Group	1, 17	0.071	.794		0.004	-
Anteriority*Group	2, 34	0.085	.834		0.005	.643
Laterality*Group	2, 34	0.165	.849		0.010	1.000
Block*Anteriority	2, 34	0.211	.811		0.012	.909
Block*Laterality	2, 34	0.320	.728		0.018	1.000
Block*Anteriority*Group	2, 34	1.137	.333		0.063	.909
Block*Laterality*Group	2, 34	1.498	.238		0.081	1.000
Anteriority*Laterality	4, 68	13.422	< .001	***	0.441	.967
Anteriority*Laterality*Group	4, 68	0.370	.829		0.021	.967
Block*Anteriority*Laterality	4, 68	0.478	.752		0.027	.913
Block*Anteriority*Laterality*Group	4, 68	0.556	.696		0.032	.913

Note: *** $p < .001$.

References

Logan, G. D. (2015). The point of no return: A fundamental limit on the ability to control thought and action. *Quarterly Journal of Experimental Psychology*, *68*(5), 833–857. https://doi.org/10.1080/17470218.2015.1008020

Pinet, S., Dell, G. S., & Alario, F.-X. (2019). Tracking Keystroke Sequences at the Cortical Level Reveals the Dynamics of Serial Order Production. *Journal of Cognitive Neuroscience*, *31*(7), 1030–1043. https://doi.org/10.1162/jocn_a_01401

Pinet, S., Hamamé, C. M., Longcamp, M., Vidal, F., & Alario, F.-X. (2015). Response planning in word typing: Evidence for inhibition. *Psychophysiology*, *52*(4), 524–531. https://doi.org/10.1111/psyp.12373

Scaltritti, M., Alario, F.-X., & Longcamp, M. (2018). The Scope of Planning Serial Actions during Typing. *Journal of Cognitive Neuroscience*, *30*(11), 1620–1629. https://doi.org/10.1162/jocn_a_01305

Sperl, L., Ambrus, G. G., Kaufmann, J. M., Schweinberger, S. R., & Cañal-Bruland, R. (in revision). Electrophysiological correlates underlying interference control in motor tasks.

Verbruggen, F., Chambers, C. D., & Logan, G. D. (2013). Fictitious Inhibitory Differences: How Skewness and Slowing Distort the Estimation of Stopping Latencies. *Psychological Science*, *24*(3), 352–362. https://doi.org/10.1177/0956797612457390

Verbruggen, F., Logan, G. D., & Stevens, M. A. (2008). STOP-IT: Windows executable software for the stop-signal paradigm. *Behavior Research Methods*, *40*(2), 479–483. https://doi.org/10.3758/BRM.40.2.479
